# Size selective recognition of small esters by a negative allosteric hemicarcerand

**DOI:** 10.3762/bjoc.6.10

**Published:** 2010-02-03

**Authors:** Holger Staats, Arne Lützen

**Affiliations:** 1 University of Bonn, Kekulé-Institute of Organic Chemistry and Biochemistry, Gerhard-Domagk-Str. 1, D-53121 Bonn, Germany

**Keywords:** allosteric receptors, 2,2′-bipyridine, hemicarcerand, molecular recognition, resorcin[4]arene

## Abstract

A bis(resorcinarene) substituted 2,2′-bipyridine was found to bind weakly to small esters like ethyl acetate whereas more bulky esters were not recognized by this hemicarcerand. This size selective molecular recognition could be controlled by a negative cooperative allosteric effect: coordination of a triscarbonyl rhenium chloride fragment to the bipyridine causes a conformational rearrangement that orientates the resorcinarene moieties in different directions so that they cannot act cooperatively in the binding of the substrate.

## Introduction

**Scheme 1:**
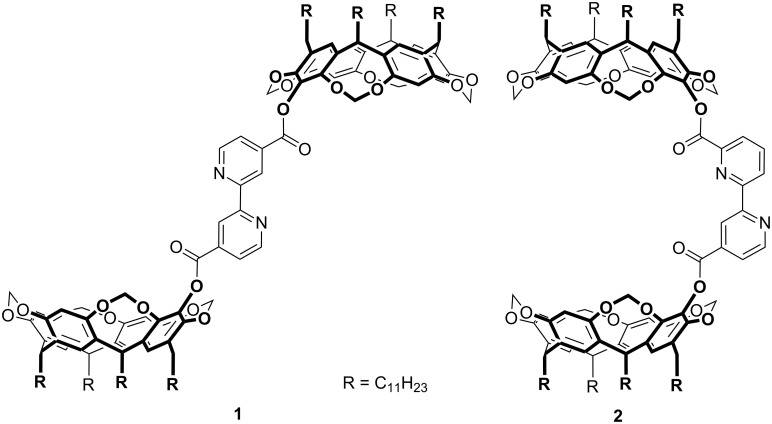
Bis(resorcinarene) esters of 4,4′- and 4,6′-(2,2′-bipyridyl)dicarboxylic acid, **1** and **2**, respectively.

Nature uses allosteric effects in a very elegant manner to control numerous biochemical pathways [1]. Thus, the transfer of this principle to artificial systems is both challenging and promising to regulate supramolecular functionality [2]. The idea is to employ cooperative effects in the selective association of more than one substrate to different binding sites of a single receptor. This causes conformational rearrangements that switch on or off a function that is inherently embedded in the different parts of the molecule but which need to be specially arranged in space in order to act in an optimized cooperative fashion. Some time ago we were able to report on a heterotropic positive cooperative allosteric analogue (**1**) [3] of some well known hemicarcerands [4–5] (Scheme 1). Their recognition behaviour towards non-polar substrates could be dramatically changed upon coordination of a late transition metal ion such as silver(I) as an effector or modulator to a central 2,2′-bipyridine. This structure has proved to be an excellent allosteric centre [6–26] due to its well defined ability to switch between *syn*- and an *anti*-conformations [27]. Recently, we were able to synthesize a number of derivatives of this first example of an allosteric hemicarcerand and their metal complexes formed upon coordination to metal salts or complexes like AgBF_4_, CuBF_4_, [Cuphen]BF_4_, or [(CO)_5_ReCl] [28]. Among these, bis(resorcin[4]arene) substituted 2,2′-bipyridine **2** is a structural isomer of **1** differing only in the substitution pattern of the central bipyridine unit: whereas in **1** the 2,2′-bipyridine is substituted in the 4,4′-position it carries the resorcinarene moieties in 4,6′-position in **2**.

This, however, causes a very important difference in the receptor’s function: **1** is an example of a receptor that can be controlled by a heterotropic positive allosteric effect because it has an open conformation in its non-coordinated form since the 2,2′-bipyridine adopts an *anti*-conformation which is inactive as a receptor. Therefore, it needs to be activated by the coordination of a transition metal ion in order to form the closed conformation where the two resorcinarene moieties can act together to bind to the substrate. **2**, however, can adopt a closed conformation that is ready to act as a receptor but can be transferred into an inactive open form upon coordination of a transition metal ion as an effector. Thus, **2** is designed to act as a first example for a heterotropic negative cooperative allosteric hemicarcerand whose function as a receptor can be switched off by adding a transition metal ion as an effector. In this account we present a proof of principle that this concept indeed works: **2** was found to have a weak affinity for simple esters in a size selective manner in the absence of an effector whereas it does not show any binding affinity when it is coordinated to a tris(carbonyl)rhenium chloride fragment – thus showing negative cooperative allosteric behaviour.

## Results and Discussion

**Scheme 2:**
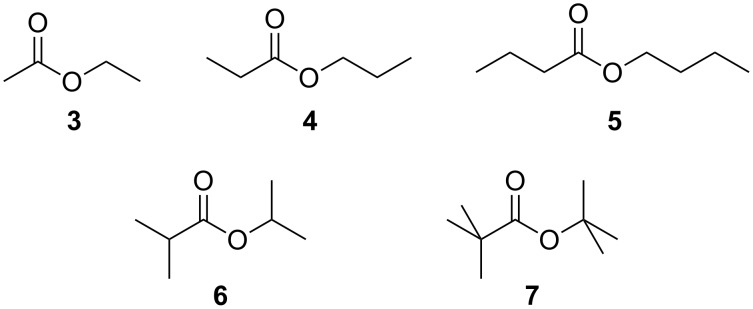
Simple esters used as model substrates in this study.

Molecular mechanics studies (MMFF force field, Spartan 08) indicate that **2** offers only a very small cavity surrounded by rather non-polar acetal and aryl groups for the encapsulation of a small non-polar substrate via dispersive interactions. Unfortunately, **2** is soluble only in rather non-polar solvents which, however, are reasonably good guests for **2** themselves if they are small enough to fit into the cavity. Moreover, they are also good solvents for any other non-polar substrate. Thus, we did not expect to observe high affinities in these binding studies. In order to minimize the competition of the substrates with the solvent for the encapsulation we chose to do the binding studies in mesitylene-*d*_12_ which seemed to be too large to fit into the cavity of **2**. We then chose to test its ability to bind to simple esters like ethyl acetate (**3**), *n*-propyl propionate (**4**), *n*-butyl butyrate (**5**), isopropyl isobutyrate (**6**), and *tert*-butyl pivalate (**7**) (Scheme 2) because esters show reasonably low polarity and can easily be obtained in different sizes and shapes.

**Figure 1:**
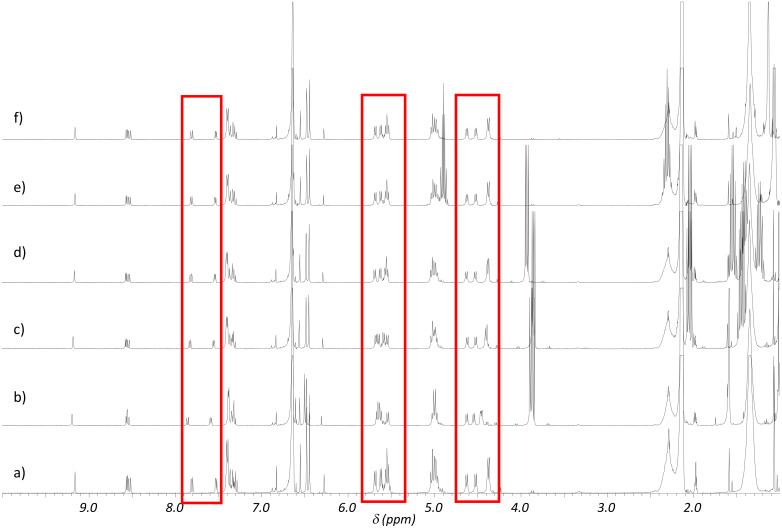
Qualitative binding studies of **2** and the model substrates **3**–**7**. ^1^H NMR spectra (500.1 MHz, 298 K in mesitylene-*d*_12_, *c*_0_(**2**) = 5 mmol/L) of a) **2**, b) **2** and 15 equiv of **3**, c) **2** and 15 equiv of **4**, d) **2** and 15 equiv of **5**, e) **2** and 15 equiv of **6**, f) **2** and 15 equiv of **7**. Marked in red rectangles are the regions of the signals of the acetal and some of the bipyridine hydrogen atoms of **2**.

With respect to the huge mass difference we decided to use an excess of guest rather than the host to get some initial qualitative information about the recognition behaviour from NMR investigations and in order to avoid solubility problems and other unspecific aggregation of **2**. Thus, in a first set of experiments 15 equivalents of the respective esters were added to a 5 mM solution of **2** in mesitylene-*d*_12_ in order to observe an effect for the signals of the bis(resorcinarene) host, whereas effects for the guests were only expected in case of slow guest exchange behaviour on the NMR timescale (Figure 1).

As expected, only the smallest esters **3** and **4** cause small but significant shifts of some of the receptor’s proton signals that can be assigned to hydrogen atoms of the acetal bridges of the resorcinarenes (4.2–4.8 and 5.3–5.9 ppm) and of the bipyridine (7.5–8.0 ppm), respectively. Note that these hydrogen atoms are all located more or less inside the cavity which clearly indicates encapsulation of the esters rather than a kind of accidental binding to the receptor’s convex outer surface or within the long alkyl chains in its periphery, whose signals remain almost unchanged.

The guest exchange, however, was found to be fast on the NMR time-scale since we could not detect different sets of signals for the encapsulated guest and the free guest but rather observed an averaged signal very close to the one of the free guest due to the large excess of the free substrate. Despite the large excess of the free guest this also hints at a rather low binding affinity of **2** towards the esters as expected for the reasons given above. Addition of the larger esters **5**–**7**, however, did not result in any significant shifts indicating size-selective discrimination of the different esters.

**Figure 2:**
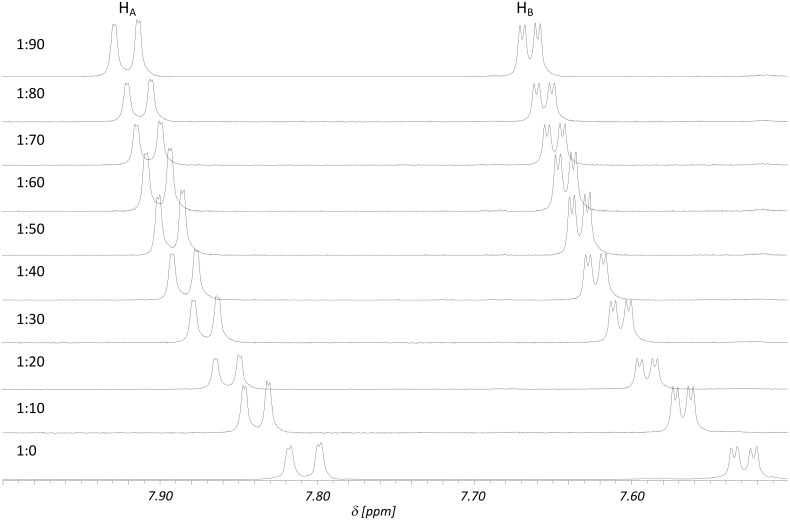
^1^H NMR titration (500.1 MHz, 298 K, *c*_0_(**2**) = 5.3 mmol/L) of **2** with increasing amounts of ethyl acetate. H_A_ and H_B_ are both signals of protons of the 2,2′-bipyridine (see Supporting Information for further details).

In order to evaluate this phenomenon further we performed an NMR titration to determine the association constant for the binding of the arguably best guest ethyl acetate assuming a 1:1 stoichiometry of the resulting host-guest complex (Figure 2).

Analysis of the binding isotherms by non-linear regression revealed only a small association constant of *K* = 9 ± 1 M^−1^ which, however, was not unexpected given the fact that binding occurs mainly due to quite weak dispersive interactions in a rather competitive solvent (for this kind of interactions).

**Scheme 3:**
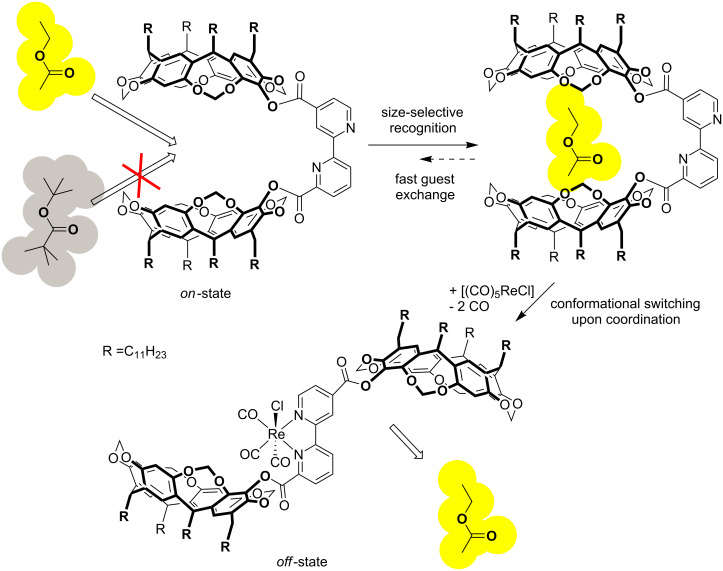
Binding model of the negative cooperative allosteric behaviour of **2**.

Having established the successful, but weak, binding in its active conformation we then examined its recognition behaviour in the presence of an effector. As demonstrated in an earlier study [28] pentacarbonylrhenium(I) chloride is able to form a stable complex [(CO)_3_Re(**2**)Cl] that was found to be soluble in mesitylene-*d*_12_. Usually, 2,2′-bipyridyl complexes of rhenium are kinetically almost inert. In this case, however, we were able to show that the rhenium can indeed be removed by adding ethylene diamine tetraacetic acid (EDTA). Thus, pentacarbonylrhenium(I) chloride seemed indeed an excellent effector here because it can be used to switch off **2** by coordination to the bipyridine and switch it on again when it is removed. When we repeated the titration with this complex we did observe some shifts of the host signals but these did not reach any saturation and the analysis of these curves did result in an association constant *K* < 1 M^−1^. This, however, indicates that the recognition behaviour of **2** can indeed be controlled in a heterotropic negative cooperative allosteric fashion (Scheme 3).

Having established this first example for a negative allosteric hemicarcerand we are now working on the improvement of the performance of our allosteric receptors, e.g. by using other cavitand-building blocks with deeper cavities.

## Experimental

Compound **2** and its complex [(CO)_3_Re(**2**)Cl] were prepared according to our recently published procedure [28]. Esters **3**–**7** were purchased in p.a. quality. Mesitylene-*d*_12_ and [(CO)_5_ReCl] were obtained form commercial sources and used as received. NMR spectra were recorded on a Bruker DRX 500 spectrometer. ^1^H NMR Chemical shifts are reported as δ values (ppm) relative to residual non-deuterated solvent as the internal standard.

Analysis of the binding isotherms obtained from the NMR titration experiments was done by non-linear regression methods.

## Supporting Information

Binding isotherms obtained from the NMR titrations.

File 1 NMR Titrations
