# Supplementary material for: Size selective recognition of small esters by a negative allosteric hemicarcerand
Source: Beilstein J Org Chem. 2010 Feb 3;6:10. doi: 10.3762/bjoc.6.10 (PMC2870999; doi:10.3762/bjoc.6.10)
Supplement: File 1 — NMR Titrations [file Beilstein_J_Org_Chem-06-10-s001.pdf]

# Supporting Information

**for**

# Synthesis of some novel annulated pyrido[2,3-*d*]pyrimidines via stereoselective intramolecular hetero Diels-Alder reactions of 1-oxa-1,3-butadienes

Mohit L. Deb and Pulak J. Bhuyan*

Address: Medicinal Chemistry Division, North East Institute of Science & Technology, Jorhat 785006, Assam, India, Fax: 0376 2370011

E-mail: Pulak J. Bhuyan - pulak_jyoti@yahoo.com

* Corresponding author

**Spectroscopic and elemental analyses data of the compounds 6b–h and 7b–e.**

***cis*-isomer 6b:** mp = 265 °C. IR (KBr); 3038, 2912, 1715, 1698, 1187 $cm^{-1}$. $^{1}H$ NMR (300 MHz, $CDCl_3$); δ, 1.73 (s, 6H), 3.10 (s, 3H), 3.31 (s, 3H), 3.43 (d, *J*=4.81 Hz, 2H), 3.50-3.59 (m, 1H), 4.19 (d, *J*=4.72 Hz, 2H), 4.33 (d, *J*=7.01 Hz, 1H), 6.58-7.21 (m, 5H), $^{13}C$ NMR (75 MHz, $CDCl_3$); δ, 168.02, 163.47, 162.16, 156.35, 149.44, 137.53, 129.41, 124.04, 120.11, 105.43, 85.97, 51.51, 37.08, 33.31, 0.05, 29.39, 28.89, 27.68. *m/z* 426.6 $(M+H)^+$. Anal. Calcd. for $C_{22}H_{23}N_3O_6$; C, 62.11; H, 5.41; N, 9.88; found C, 62.03; H, 5.60; N, 10.15.

***trans*-isomer 7b:** mp = 260-261 °C. IR (KBr); 3038, 2912, 1715, 1698, 1187 $cm^{-1}$. $^{1}H$ NMR (300 MHz, $CDCl_3$); δ, 1.73 (s, 6H), 3.10 (s, 3H), 3.31 (s, 3H), 3.44 (d, *J*=4.93 Hz, 2H), 3.50-3.61 (m, 1H), 4.17 (d, *J*=4.72 Hz, 2H), 4.39 (d, *J*=13.69 Hz, 1H), 6.59-7.21 (m, 5H), $^{13}C$ NMR (75 MHz, $CDCl_3$); δ, 168.02, 163.44, 162.11, 154.89, 149.71, 137.53, 129.41, 124.04, 120.11, 105.43, 85.97, 51.77, 37.08, 33.47, 30.11, 29.39, 28.89, 27.68. *m/z* 426.6 $(M + H)^+$. Anal. Calcd. for $C_{22}H_{23}N_3O_6$; C, 62.11; H, 5.41; N, 9.88; found C, 62.21; H, 5.58; N, 10.08.

***cis*-isomer 6c:** mp = 233-235 °C. IR (KBr); 2933, 1698, 1679, 1168 $cm^{-1}$. $^{1}H$ NMR (300 MHz, $CDCl_3$); δ, 2.86 (s, 3H), 2.96 (s, 3H), 2.98 (s, 3H), 3.12 (d, *J*=6.60 Hz, 2H), 3.28 (s, 6H), 3.31-3.42 (m, 1H), 4.02 (d, *J*=4.9 Hz, 2H), 4.23 (d, *J*=5.94 Hz, 1H). $^{13}C$ NMR (75 MHz, $CDCl_3$); δ, 163.20, 156.06, 153.44, 151.21, 101.22, 89.61, 53.12, 37.79, 34.36, 31.75, 29.06, 28.78, 28.31, 25.61. *m/z* 376.5 $(M + H)^+$. Anal. Calcd. for $C_{17}H_{21}N_5O_5$; C, 54.40; H, 5.60; N, 18.66; found C, 54.53; H, 5.47; N, 18.54.

***trans*-isomer 7c:** mp = 241-243 °C. IR (KBr); 2933, 1698, 1679, 1168 $cm^{-1}$. $^{1}H$ NMR (300 MHz, $CDCl_3$); δ, 2.83 (s, 3H), 2.96 (s, 3H), 2.98 (s, 3H), 3.12 (d, *J*=6.60 Hz, 2H), 3.26 (s, 6H), 3.31-3.40 (m, 1H), 4.07 (d, *J*=4.71 Hz, 2H), 4.21 (d, *J*=14.35 Hz, 1H). $^{13}C$ NMR (75 MHz, $CDCl_3$); δ, 163.41, 156.09, 153.47, 151.21, 101.71, 89.38, 53.12, 37.44, 34.30, 31.71, 29.80, 28.79, 28.39, 25.21. *m/z* 374.3 $(M - H)^+$. Anal. Calcd. for $C_{17}H_{21}N_5O_5$; C, 54.40; H, 5.60; N, 18.66; found C, 54.57; H, 5.69; N, 18.57.

***cis*-isomer 6d:** mp = 224-227 °C. IR (KBr); 2923, 1717, 1698, 1186 $cm^{-1}$. $^{1}H$ NMR (300 MHz, $CDCl_3$); δ, 1.73 (s, 6H), 2.86 (s, 3H), 2.98 (s, 3H), 3.12 (d, *J*=6.60 Hz, 2H), 3.17 (s, 3H), 3.31-3.40 (m, 1H), 4.06 (d, *J*=4.88 Hz, 2H), 4.33 (d, *J*=6.41 Hz, 1H), $^{13}C$ NMR (75 MHz, $CDCl_3$); δ, 168.02, 163.47, 162.32, 154.81, 149.42, 137.50, 101.02, 78.46, 51.51, 37.08, 34.36, 32.03, 30.05, 29.39, 28.89, 27.68. *m/z* 364.7 $(M + H)^+$. Anal. Calcd. for $C_{17}H_{21}N_3O_6$; C, 56.19; H, 5.78; N, 11.57; found C, 56.07; H, 5.64; N, 11.71.

***trans*-isomer 7d:** mp = 231-233 °C. IR (KBr); 2923, 1717, 1698, 1186 $cm^{-1}$. $^{1}H$ NMR (300 MHz, $CDCl_3$); δ, 1.73 (s, 6H), 2.83 (s, 3H), 2.98 (s, 3H), 3.10 (d, *J*=4.58 Hz, 2H), 3.17 (s, 3H), 3.33-3.41 (m, 1H), 4.06 (d, *J*=4.94 Hz, 2H), 4.31 (d, *J*=15.22 Hz, 1H), $^{13}C$ NMR (75 MHz, $CDCl_3$); δ, 167.87, 163.47, 162.43, 154.09, 149.47, 137.50, 101.98, 78.93, 51.54, 37.08, 34.36, 32.11, 30.08, 29.39, 28.89, 27.68. *m/z* 364.7 $(M + H)^+$. Anal. Calcd. for $C_{17}H_{21}N_3O_6$; C, 56.19; H, 5.78; N, 11.57; found C, 56.11; H, 5.91; N, 11.69.

***cis*-isomer 6e:** mp = 248-249 °C. IR (KBr); 3031, 2953, 1698, 1166 $cm^{-1}$. $^{1}H$ NMR (300 MHz, $CDCl_3$); δ, 2.93 (s, 6H), 3.10 (s, 3H), 3.19 (s, 3H), 3.24-3.41 (m, 3H), 3.86 (s, 2H), 4.17 (d, *J*=3.96 Hz, 2H), 4.36 (d, *J*=7.76 Hz, 1H), 6.89-7.21 (m, 5H). $^{13}C$ NMR (75 MHz, $CDCl_3$); δ, 163.27, 156.33, 153.34, 149.71, 131.73, 128.48, 128.19, 122.56, 118.10, 101.32, 78.28, 51.52, 44.05, 36.44, 32.05, 29.13, 28.78, 28.41, 26.18. *m/z* 452.4 $(M + H)^+$. Anal. Calcd. for $C_{23}H_{25}N_5O_5$; C, 61.19; H, 5.54; N, 15.52; found C, 61.33; H, 5.41; N, 15.36.

***trans-i*somer 7e:** mp = 243-244 °C. IR (KBr); 3031, 2953, 1698, 1166 $cm^{-1}$. $^{1}H$ NMR (300 MHz, $CDCl_3$); δ, 2.96 (s, 6H), 3.12 (s, 3H), 3.16 (s, 3H), 3.24-3.41 (m, 3H), 3.86 (s, 2H), 4.13 (d, *J*=4.32 Hz, 2H), 4.34 (d, *J*=13.59 Hz, 1H), 6.92-7.18 (m, 5H). $^{13}C$ NMR (75 MHz, $CDCl_3$); δ, 163.25, 156.41, 153.34, 149.77, 131.73, 128.48, 128.13, 121.67, 118.10, 101.49, 79.02, 51.57, 44.05, 37.39, 32.11, 29.13, 28.78, 28.37, 26.43. *m/z* 474.4 $(M + Na)^+$. Anal. Calcd. for $C_{23}H_{25}N_5O_5$; C, 61.19; H, 5.54; N, 15.52; found C, 61.27; H, 5.65; N, 15.44.

***cis*-isomer 6f:** mp = 257-259 °C. IR (KBr); 3041, 2953, 1698, 1171 $cm^{-1}$. $^{1}H$ NMR (300 MHz, $CDCl_3$); δ, 1.28-1.34 (m, 6H), 2.96 (s, 3H), 2.98 (s, 3H), 3.21-3.27 (m, 4H), 3.61-3.66 (m, 3H), 4.24 (d, *J*=5.12 Hz, 2H), 4.36 (d, *J*=5.02 Hz, 1H), 6.98-7.23 (m, 5H). $^{13}C$ NMR (75 MHz, $CDCl_3$); δ, 162.70, 161.94, 156.38, 153.82, 153.37, 149.81, 135.42, 129.95, 123.86, 118.59, 104.34, 89.61, 51.55, 37.05, 34.13, 34.01, 31.81, 28.78, 28.31, 26.18, 12.34, 12.09. *m/z* 466.5 $(M + H)^{+}$. Anal. Calcd. for $C_{24}H_{27}N_5O_5$; C, 61.93; H, 5.80; N, 15.05; found C, 61.82; H, 5.71; N, 15.23.

***cis*-isomer 6g:** mp = 267-269 °C. IR (KBr); 2953, 1698, 1686, 1168 $cm^{-1}$. $^{1}H$ NMR (300 MHz, $CDCl_3$); δ, 1.26-1.33 (m, 6H), 2.86 (s, 3H), 2.96 (s, 3H), 2.98 (s, 3H), 3.02 (d, *J*=5.44 Hz, 2H), 3.21-3.27 (m, 4H), 3.31-3.42 (m, 1H), 4.02 (d, *J*=5.04 Hz, 2H), 4.21 (d, *J*=5.78 Hz, 1H). $^{13}C$ NMR (75 MHz, $CDCl_3$); δ, 162.28, 161.47, 156.06, 153.73, 153.22, 151.33, 101.22, 77.83, 53.97, 37.71, 34.36, 34.13, 34.0, 33.02, 28.78, 28.31, 25.61, 12.34, 12.09. *m/z* 404.8 $(M + H)^{+}$. Anal. Calcd. for $C_{19}H_{25}N_5O_5$; C, 56.57; H, 6.20; N, 17.36; found C, 56.68; H, 6.11; N, 17.53.

***cis*-isomer 6h:** mp = 274-277 °C. IR (KBr); 3041, 2947, 1698, 1166 $cm^{-1}$. $^{1}H$ NMR (300 MHz, $CDCl_3$); δ, 1.26-1.31 (m, 6H), 2.96 (s, 3H), 2.98 (s, 3H), 3.08 (d, *J*=5.89 Hz, 2H), 3.21-3.27 (m, 4H), 3.33-3.41 (m, 1H), 3.86 (s, 2H), 4.21 (d, *J*=5.12 Hz, 2H), 4.32 (d, *J*=7.87 Hz, 1H), 6.93-7.18 (m, 5H). $^{13}C$ NMR (75 MHz, $CDCl_3$); δ, 163.07, 161.93, 156.61, 153.81, 153.47, 151.38, 131.73, 128.48, 122.56, 118.10, 101.12, 78.21, 51.52, 44.31, 37.09, 34.35, 34.13, 32.23, 28.78, 28.41, 26.18, 12.34, 12.10. *m/z* 478.6 $(M - H)^{+}$. Anal. Calcd. for $C_{25}H_{29}N_5O_5$; C, 62.63; H, 6.05; N, 14.61; found C, 62.74; H, 6.17; N, 14.37.
